# P-1965. A Smart Way of Chart Review for Research: Utilizing Large Language Models (LLM) for Extraction of Unstructured Text Data

**DOI:** 10.1093/ofid/ofaf695.2132

**Published:** 2026-01-11

**Authors:** Ali Ejaz, Jeffrey Shu, Jarrod Dalton, Abhishek Deshpande, Ken Koon Wong

**Affiliations:** Cleveland Clinic Akron General, Akron, OH; Cleveland Clinic Learner College of Medicine of Case Western reserve University School of Medicine, Cleveland, Ohio; Cleveland Clinic Learner College of Medicine of Case Western reserve University School of Medicine, Cleveland, Ohio; Cleveland Clinic, Cleveland, Ohio; Cleveland Clinic, Cleveland, Ohio

## Abstract

**Background:**

Large Language Models (LLMs) have potential to improve clinical research, particularly in parsing unstructured data. Stool culture reports, typically recorded as unstructured free text, pose challenges for systematically identifying positive and negative pathogens and may require hours of manual chart review to extract relevant data fields. This limits the utilization of microbiology reports for quality improvement projects, clinical research, and public health reporting. This study evaluates the use of LLMs to transform free-text stool culture reports into structured, tabular data for large-scale analyses.

**Figure 1.**
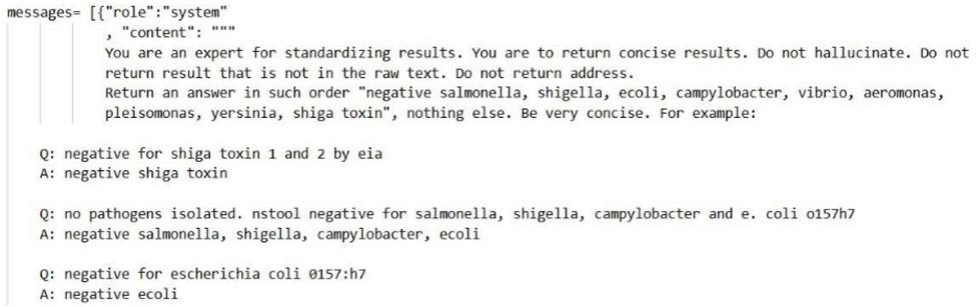
Prompt Engineering (Few Shot Prompt)

**Figure 2:**
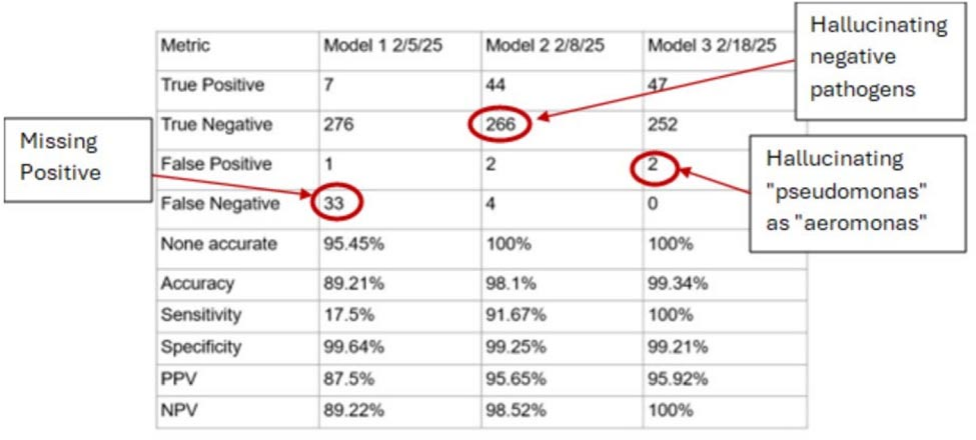
Performance metrics for all 3 iterations

**Methods:**

We conducted a retrospective cohort study of stool culture reports (2010–2024) from 12 acute-care hospitals in the Cleveland Clinic Health System. (IRB 24-651) The GPT4o-mini model via Python 3.11 OpenAI module was utilized on a HIPAA-compliant Azure Cloud framework. Few-shot prompt engineering was used to instruct the LLM to return a binary result and pathogen name. Model performance was evaluated on a 10% random subset. Prompts were iteratively updated to optimize performance and improve accuracy. A simplified estimation calculated the time saved using LLMs compared to manual chart review.

**Results:**

A total of 65,703 stool culture results comprised of 3270 unique text strings were included in analysis. A total of 3 iterations were performed, requiring 30 minutes per iteration and a total operating cost of $2.14. (Figure 1) All performance metrics improved with iterative changes to the prompt, with accuracy improving from 89.21% to 99.34%. (Figure 2) Overall, we estimate 12.1 hours of time and cognitive effort saved using an LLM approach compared to traditional chart review (1.5 vs 13.6 hours).

**Conclusion:**

LLMs offer a scalable way to extract structured data from unstructured free text. In our use-case with stool culture reports, we demonstrate that LLMs significantly reduced time, cognitive effort, and costs associated with data extraction and data cleaning. However, hallucinations remain a challenge in LLM approaches, though future advances could mitigate this limitation. Future work could expand LLM applications to other data types including clinical notes and polymicrobial culture reports.

**Disclosures:**

All Authors: No reported disclosures

